# Cancer-Associated Thrombosis in Patients Treated with Immune Checkpoint Inhibitors

**DOI:** 10.3390/ijms27041874

**Published:** 2026-02-15

**Authors:** Alice Ilari, Maria Ida Abbate, Melina Verso, Mara Graziani, Pietro Cafaro, Luca Sala, Francesca Colonese, Diego Luigi Cortinovis, Stefania Canova

**Affiliations:** 1 Medical Oncology Unit, IRCCS Fondazione San Gerardo dei Tintori, 20900 Monza, Italy; alice.ilari@irccs-sangerardo.it (A.I.); mariaida.abbate@irccs-sangerardo.it (M.I.A.); pietro.cafaro@irccs-sangerardo.it (P.C.); luca.sala@irccs-sangerardo.it (L.S.); francesca.colonese@irccs-sangerardo.it (F.C.); diegoluigi.cortinovis@irccs-sangerardo.it (D.L.C.); 2 Department of Clinical Medicine, University of Milano-Bicocca, 20900 Monza, Italy; 3 Internal, Vascular and Emergency Medicine—Stroke Unit, Department of Medicine and Surgery, University of Perugia, 06123 Perugia, Italy; melina.verso@unipg.it (M.V.); maragraziani11@gmail.com (M.G.); 4 Medicine and Surgery Department, University of Brescia, 25121 Brescia, Italy

**Keywords:** cancer-associated thrombosis, venous thromboembolism, immune checkpoint inhibitors, risk assessment models, biomarkers

## Abstract

The incidence of cancer-associated thrombosis has increased in recent years. While the association between venous thromboembolism (VTE) and chemotherapy is well established, there is no clear link between immune checkpoint inhibitors (ICIs) and VTE risk. Many risk assessment models (RAMs) have been developed to identify high-risk patients who need prophylaxis. However, these models are validated in patients undergoing chemotherapy, while they are scarce in those receiving immunotherapy. Moreover, the mechanisms linking ICIs to thrombosis are still a matter of debate. They include the upregulation of pro-inflammatory intracellular pathways, the release of cytokines, the activation of innate immune cells, the release of tissue factors and platelet activation, and the increase in adhesion molecules, thus resulting in the recruitment of agents involved in coagulation. Promising biomarkers are emerging to identify patients undergoing ICIs who are at high risk of developing VTE and need prophylaxis. In this review we investigate the possible causation between cancer-associated thrombosis (CAT) and immunotherapy and the underlying pathophysiological mechanisms. Thus, we suggest the most appropriate therapeutic approaches based on currently available data.

## 1. Introduction

Cancer-associated thrombosis (CAT) refers to venous thromboembolism (VTE) that occurs in the context of cancer, either as a result of the malignancy itself or as a consequence of anticancer therapy. CAT encompasses thrombotic events such as pulmonary embolism (PE), deep vein thrombosis of the lower limbs (LL-DVT) or upper extremities (UE-DVT), and, in some cases, thrombosis of the splanchnic veins or cerebral venous sinuses. In addition to venous events, cancer patients are also at increased risk for arterial thromboembolic events (ATEs), which can manifest as ischemic strokes, acute coronary syndromes, or peripheral and visceral arterial thrombosis.

In patients with active cancer, VTE occurs at an estimated rate of 18.2 events per 100 patient-years and represents the second leading cause of death, after cancer progression. In recent decades, the reported incidence of CAT has steadily increased. This trend is likely driven by enhanced clinical awareness, improved diagnostic imaging techniques, advances in anticancer therapies, and improvements in the overall survival of cancer patients [1,2].

VTE in cancer patients is associated with greater morbidity, which can lead to a decline in the quality of life, interrupt treatment regimes, and cause elevated mortality rates. Moreover, thromboembolic events are associated with worse survival rates [3,4,5].

While the association between VTE and chemotherapy is well established, there is no clear link between immune checkpoint inhibitors (ICIs) and VTE risk. Moreover, there are limited data available due to the underestimation of events and unreported pharmacovigilance data.

The aim of this narrative review is to investigate the possible association between CAT and immunotherapy and the underlying pathophysiological mechanisms and to suggest the most appropriate therapeutic approaches based on currently available data. We conducted a search of the published literature from 2011 to 2025.

## 2. Methods

### 2.1. Literature Search Strategy

A structured literature search was conducted using the PubMed and Scopus databases to identify studies investigating CAT in patients treated with ICIs. The search included articles published between 2004 and 2025 and was restricted to English-language, peer-reviewed publications involving adult patients.

The search strategy combined cancer- and thrombosis-related terms with treatment-related keywords using Boolean operators, including: “cancer” OR “tumor” AND “thrombosis” OR “cancer-associated thrombosis” OR “venous thromboembolism” OR “venous thromboembolic events” OR “arterial thromboembolic events” OR “thrombotic disease” OR “cardiovascular events” OR “cardiovascular toxicity” AND “immune checkpoint inhibitors” OR “immunotherapy” OR “chemotherapy”.

### 2.2. Eligibility Criteria

*Inclusion criteria were:*

- Controlled and uncontrolled clinical trials, observational studies, and cohort studies;
- Studies reporting venous and/or arterial thromboembolic events in patients with cancer;
- Relevant translational or mechanistic studies providing biological insights into CAT in the context of immunotherapy.

*Exclusion c* *riteria were:*

- Conference abstracts without full-text availability;
- Studies conducted exclusively in pediatric populations;
- Articles not reporting thrombotic outcomes or not relevant to CAT;
- Non-English publications.

### 2.3. Study Selection and Limitations

The decision to include or exclude studies was made by the authors through discussion and consensus. Given the narrative nature of the review and the heterogeneity of study designs and reported outcomes, no formal quality assessment tool was applied. We acknowledge that this approach may have resulted in a biased representation of the available evidence.

## 3. Understanding CAT

The pathogenesis of thrombosis in patients with malignant tumors is complex, involving various factors ranging from clinical and histopathological to genetic influences. Specifically, blood hypercoagulability includes the release of procoagulant substances by tumor cells, the interaction between tumor cells and the fibrinolytic system, tumor cell-mediated platelet activation, tumor-associated complement activation, and the interaction between tumor cells and inflammatory pathways [6].

Patient-related factors may also contribute to the risk of CAT, with a prior history of VTE being the most significant clinical predictor (currently 2- to 8-fold increased risk). Additional factors, such as old age, male gender, high body mass index, and recent hospitalization, are associated with a slight but consistent increase in risk (approximately 1.2-fold). Conversely, the comorbidity burden and socio-economic deprivation have not been shown to be independently associated with CAT in contemporary cohort studies [7,8].

Among cancer characteristics, the type of malignancy plays a pivotal role in determining the risk of CAT. Epidemiological data consistently show that certain tumor types are associated with a markedly higher thrombotic risk. For instance, hematologic cancers, particularly acute leukemia, exhibit the highest 12-month incidence of VTE (up to 18.6%) and the highest odds ratio (OR) for hospital-associated VTE diagnosis (OR 26.2). Lung cancer also carries a substantially elevated risk, with an OR of 24.8 and a 12-month incidence of VTE ranging from 6% to 10% in population-based studies. Similarly, gastrointestinal malignancies—including pancreatic, gastric, and esophageal cancers—consistently rank among the highest-risk groups, with ORs up to 18.9 and cumulative incidence rates of around 8–10% [9]. Brain tumors are also notable, with a high VTE risk (OR 8.0), which is potentially due to neurological impairment and immobility. By contrast, cancers such as prostate, breast, and gynecological tumors (other than ovarian cancer) tend to show lower—though still clinically relevant—risks, with ORs ranging between 2.3 and 3.5 [10,11].

Beyond cancer histology, tumor stage and treatment type may also influence the risk of CAT. Advanced-stage disease is independently associated with a higher thrombotic burden: stage III and stage IV cancers confer approximately 2- and 4-fold increased risks, respectively, compared to early-stage tumors [12].

Finally, although cancer treatment also plays a role, its impact on thrombotic risk is not fully defined. Patients who undergo anticancer treatment, such as cytotoxic chemotherapies, ICIs, and targeted or endocrine therapies, are associated with a 1.2- to 1.5-fold increase in VTE risk as compared with patients who do not undergo the treatment.

ICIs represent a class of drugs that block the intracellular signaling involved in regulating physiological immune responses to cancer, resulting in a strong and sustained inflammatory response. These drugs target, among other things, programmed death 1 (PD-1) and its ligand (PD-L1), which lead to impaired T-cell-mediated antitumor immunity. By inhibiting the PD-1/PD-L1 axis, the physiological anticancer immune response is reinvigorated. Another class of ICIs is represented by cytotoxic T-lymphocyte-associated protein 4 (CTLA-4) inhibitors. CTLA-4 is a key regulator of the interaction between antigen-presenting cells (APCs) and T-cells. Targeting the PD-1/PD-L1 axis and CTLA4 has revolutionized cancer therapy, leading to improvements in treatment efficacy and patient survival rates, even in cases of advanced cancer patients. However, the widespread use of ICIs in oncology has raised concerns about their potential to cause thromboembolic events (TEEs). Although they are often overshadowed by other immune-related adverse events (irAEs), such as colitis or pneumonitis, thrombotic complications—including VTE and ATEs—are emerging as clinically relevant and potentially fatal toxicities. Recent evidence suggests that TEEs may be as severe as other grade 3/4 adverse events, particularly PE and myocardial infarction [13].

An early systematic review and meta-analysis of 68 studies (18 retrospective studies and predominantly clinical trials; total N = 20,273) reported pooled incidences of 1.1% (95% CI 0.65–1.45) for ATEs and 2.7% (95% CI 1.4–5.4) for VTE among ICI-treated patients [3]. A subsequent meta-analysis of RCTs found no increased VTE risk in ICI users compared with non-ICI controls (OR 0.99; 95% CI 0.82–1.19) [14].

However, estimates derived from randomized trials should be interpreted cautiously, as thrombotic outcomes may be incompletely captured owing to non-systematic screening, heterogeneous adverse-event reporting, and limited follow-up.

In contrast, post-marketing and real-world cohort studies suggest a substantially higher absolute burden of TEEs during ICI therapy. Large retrospective cohorts have reported VTE incidences ranging from 7% to 13% within the first year. In a cohort of 2854 patients, VTE rates were 7.4% at 6 months and 13.8% at 12 months, with a nearly 5-fold increase in risk after ICI initiation (HR 4.98; 95% CI 3.65–8.59) [5].

Population-based studies generally report lower incidences, with 6-month VTE rates of approximately 2–4% and 12-month rates of 4–7%, likely reflecting differences in outcome ascertainment using administrative codes rather than detailed chart review [15].

Overall, these data indicate that although TEEs are frequent during ICI therapy, current evidence does not conclusively demonstrate an ICI-specific increase in thrombotic risk beyond that attributable to cancer and systemic anticancer treatment. The wide variability in reported incidence reflects heterogeneity in study design, populations, follow-up duration, and outcome definitions.

Comparative data across ICI classes remain limited and largely indirect. No head-to-head trials have been designed to compare thrombotic risk between ICI classes, and available real-world data are confounded by tumor type, disease stage, and treatment indication.

Nevertheless, emerging comparative analyses provide hypothesis-generating signals. In a large observational study, PD-L1 monotherapy was associated with a lower VTE hazard compared with PD-1 monotherapy (HR 0.79; 95% CI 0.63–0.99), whereas dual CTLA-4/PD-1 blockade (ipilimumab plus nivolumab) was associated with a higher VTE hazard (HR 1.43; 95% CI 1.12–1.84) [5].

Reported cumulative risk estimates in cohorts of ICI-treated patients range from 9% to 24% for VTE and 2% to 5% for ATEs. Recent observational studies have reported a 2- to 4-fold increased risk of VTE during ICI therapy compared to the pre-treatment period. Furthermore, a 5-fold increase in the risk of cardiovascular events was reported within two years following ICI initiation compared to the two years prior to therapy [4,6,16,17,18,19,20,21].

The wide range of incidence may be due to the different representation of primary tumors within various studies. Higher incidence is observed in stage IV disease, lung cancer and melanoma patients, and patients treated with either double ICIs or ICI plus chemotherapy.

A large network meta-analysis of 83 randomized trials involving over 54,000 patients provides the most comprehensive assessment to date of the risk of TTEs across various ICI regimens. Monotherapy with PD-1 inhibitors—especially nivolumab—was associated with a lower overall risk of TEEs. In contrast, ipilimumab monotherapy (a CTLA-4 inhibitor) was associated with the highest incidence of VTE, particularly in patients affected by melanoma. Combination therapies, particularly dual ICIs plus chemotherapy, were associated with the highest VTE incidence, with lung cancer patients showing the greatest vulnerability. Notably, adding a single ICI to chemotherapy appeared to reduce arterial events [22]. The lower ATE rate with a single ICI plus chemotherapy may be driven by indirect comparisons versus chemotherapy alone. Moreover, estimates were imprecise given infrequent reporting and heterogeneous outcome definitions. Apparent differences may, therefore, reflect incomplete reporting of events, competing mortality, baseline cardiovascular risk imbalance, and differential exposure time rather than a true treatment effect. Taken together, any “protective” interpretation for ATE when adding a single ICI to chemotherapy should be regarded as hypothesis-generating. Mechanistic explanations—such as attenuation of immune-mediated vascular inflammation by cytotoxic chemotherapy—remain speculative and require confirmation in studies with prospective, standardized cardiovascular endpoint assessment.

ICIs are transforming cancer care, and their use will continue to expand. Real-world data indicate a substantial burden of VTE and ATE during ICI therapy, yet it remains unclear whether this reflects an ICI-specific effect, differences versus chemotherapy, or longer exposure due to improved survival.

Taken together, these factors (i.e., patient characteristics, cancer type, disease stage, and treatment modality) should be jointly considered when assessing thrombotic risk in oncology patients.

## 4. Strengths and Pitfalls of Available Risk Assessment Models (RAMs)

Due to the multiple risk factors of CAT, researchers have striven to distinguish low-risk from high-risk patients, in whom thromboprophylaxis with low-molecular-weight heparin (LMWH) or oral anticoagulants (OACs) reduces CAT events by almost 50% [23]. Over the years, many risk assessment models (RAMs) have been developed to define the risk of CAT and thus identify high-risk patients who need prophylaxis. RAMs are distinguished by tumor type and other parameters used to stratify risk, as shown in Table 1.

RAMs are mostly based on the Khorana risk score (KRS), which stratifies patients undergoing chemotherapy at low and high risk of VTE in a 6-month period [24]. The authors concluded that only high-risk patients could benefit from thromboprophylaxis to reduce the risk of VTE. Numerous validation studies have been produced, and the KRS is one of the main RAMs recommended by guidelines in daily clinical practice [25,26,27,28]. However, in the high-risk group, lung cancer, urothelial cancer, hepatocarcinoma and hematological malignancies appear to have a lower risk of VTE than other types of cancer, such as pancreatic and other gastrointestinal cancers; thus, the KRS seems to be less representative in this type of patient. Furthermore, the KRS is structured to identify the benefit of thromboprophylaxis in high-risk cancer patients who make up 25% of the population analyzed. This means that a large number of patients in the intermediate and low-risk groups who could experience VTE will not be identified and would not benefit from thromboprophylaxis [29].

Another KRS-based RAM was created by the Vienna-Cancer and Thrombosis Study (Vienna-CATS) group in 2010 [30]. The authors adopted the Khorana parameters in addition to soluble P-selectin (sP-selectin) and D-dimer. They showed that these two biomarkers can improve VTE risk prediction.

The same study group also later developed the New Vienna CATS score in 2018 [31,32]. This new predictive score has reduced the previous model to a two-factor RAM including D-dimer and the tumor site. The score stratifies the risk of VTE in cancer patients, but the role of D-dimer is still unclear. Indeed, the identification of a common standard cut-off is still absent [33].

The Vienna-CATS score was recently submitted for external validation and peer review to identify high-risk VTE patients in the first 6 months after diagnosis [34]. It has been prospectively and externally validated in a contemporary cohort of 598 ambulatory patients with solid tumors (68% stage IV disease) receiving combined anticancer therapies, including ICIs in nearly one-third of cases. The model, based on tumor type (29.1% were lung cancer patients; other main cancer types: breast, head and neck, and pancreatic) and continuous D-dimer levels, was assessed longitudinally at baseline, 3 weeks, and 3 months from starting treatment for the prediction of 6-month VTE. The cumulative 6-month VTE incidence was 9.2%, with stable discrimination across time points (overall c-statistic, 0.68). Using an 8% predicted risk threshold, the score identified a high-risk group with 15% VTE incidence and outperformed the Khorana score, which showed poor discrimination (c-statistic, 0.56) and failed to meaningfully separate low- and high-risk groups (8.4% vs. 10.7% VTE at 6 months). Although earlier versions incorporated sP-selectin, its limited assay availability and lack of standardization currently restrict routine clinical implementation, whereas D-dimer is widely accessible.

The KRS was also implemented in the PROTECHT score by adding platinum-based and/or gemcitabine chemotherapy regimens [35,36]. The performance was validated by multiple comparison trials, suggesting that this RAM distinguishes low-risk from high-risk patients better than the KRS, despite the 3-point threshold performance being suboptimal [37,38].

One of the most recent RAMs is the Italian ONKOTEV score, a KRS-based tool that includes clinical and medical history features [39]. This score has been well validated through retrospective studies, and it stratifies the target patient group better than the KRS over a 6-month VTE risk; however, it shares the same limitations of the KRS in stratification of the low-risk cohort [38].

In breast, colorectal, lung and ovarian cancer, the Comparison of Methods for Thromboembolic Risk Assessment with Clinical Perceptions and AwareneSS in Cancer-Associated Thrombosis (COMPASS-CAT) RAM was developed to stratify risk for VTE before and after initiation of chemotherapy, splitting patients into two groups: low/intermediate-risk and high-risk [40].

As stated earlier, this RAM was validated in multiple studies, in which it was shown to have good predictive value, mostly in lung cancer, in a small cohort study [41]. Thus, larger and more statistically robust cohorts in studies are needed to provide greater confidence [42].

The MD Anderson Cancer Center CAT Model (MDACC-CAT) is a multivariate analysis used to construct a nomogram CAT prediction score with good discrimination, but it lacks external validation [43]. The first score to introduce genetic risk factors in a CAT-RAM was the TiC-Onco Risk Score [44]. This score has better predictive value than the KRS, but again, it lacks validation and the disparities in the diagnostic capabilities of genetic laboratories make it a poor choice in real-life settings.

However, the high predictive power of CAT-RAM in stratifying patients into low- and high-risk groups still needs to be explored in patients undergoing ICIs. Only a few studies have investigated the use of the KRS in patients with non-small-cell lung cancer (NSCLC) who were undergoing chemotherapy and/or ICIs. A retrospective cohort study showed higher VTE rates among NSCLC patients being treated with chemotherapy than those treated with ICI alone. However, the KRS did not identify high-risk patients among those treated with immunotherapy [45]. Compared to the KRS, another study suggested that COMPASS-CAT was more predictive in identifying high-risk NSCLC patients with higher VTE rates among patients with adenocarcinoma, metastatic disease and treated with immunotherapy [46]. A retrospective study of a cohort of gastric cancer patients undergoing ICIs showed that the KRS was effective in stratifying those patients into VTE high-risk and intermediate-risk groups [47]. However, this study has several limitations, such as the potential high competitive VTE mortality risk proportional to the risk class of the KRS and a lack of potentially useful characteristics (number of ICI cycles; differences in therapies with patients undergoing platinum-based chemotherapy in high- and intermediate risk) due to the large real-world database analysis used. Conversely, the L2HSDK score is a risk assessment tool developed to predict VTE in Asian cancer patients undergoing treatment with ICIs [48]. This score uses clinical factors (liver cancer, smoking, diabetes mellitus, liver dysfunction, cardiovascular history, and KRS ≥ 3) to stratify patients in low- (score ≤ 2), moderate- (score 3–5), and high-risk (score ≥ 6) of VTE. After a 12-month follow-up, the high-risk group was found to have approximately 15 times the VTE risk compared to the low-risk group. However, this study has some limitations: the cohort was all Asian, had a higher proportion of patients with liver cancer, and lacked external validation. Moreover, the Khorana score was incorporated into the L2HSDK model as a binary variable (≥3), emerging as a strong independent predictor of VTE. Although the authors report the superior predictive accuracy of the L2HSDK score compared with the Khorana score, no direct head-to-head comparison using standardized discrimination metrics was performed.

In conclusion, CAT-RAMs are validated tools to predict VTE risk in patients undergoing chemotherapy who can benefit from thromboprophylaxis. Nevertheless, the increasing use of immunotherapy in many cancer types has not been studied in sufficient depth to stratify patients in relation to CAT risk. Thus, the understanding of mechanisms linking ICIs and CAT and the development of an ICI-specific risk model are crucial. More studies are needed to assess the correct VTE risk in cancer patients undergoing ICI treatments, and the use of specific RAMs should be part of everyday practice to complete the correct VTE risk assessment of cancer patients.

## 5. Potential Mechanisms Linking Immune Checkpoint Inhibitors (ICIs) and Thrombosis

The molecular mechanisms involved in immunotherapy and the resulting side effects are not yet fully understood. In particular, thrombotic phenomena induced by the use of ICIs are becoming increasingly relevant from a clinical point of view. An increase in thrombotic phenomena, both ATEs and VTE, has been demonstrated in patients treated with immunotherapies, especially in combination.

Numerous preclinical and clinical studies demonstrate a correlation between the use of ICIs and thrombotic risk. These studies suggest that the thrombotic process induced by the use of ICIs has a complex and multifactorial pathogenesis, in which the immune system, enhanced by the pharmacological action, loses its physiological homeostasis. The action of ICIs results in the hyperactivation of effector T lymphocytes, which release large numbers of pro-inflammatory cytokines that create a systemically inflammatory environment.

The persistent inflammatory state secondarily determines the deregulation of innate immunity with the involvement of Myeloid-Derived Suppressor Cells (MDSCs), neutrophils, and monocytes/macrophages.

This cascade triggers a vicious inflammatory–prothrombotic cycle, in which the activation of both adaptive and innate immune cells associated with the activation of the endothelium promotes thrombotic formation [49,50].

### 5.1. Lymphocyte Activation and Cytokine Response to ICIs

ICIs work by inhibiting immune checkpoints that control key processes in the immune system, such as PD-1/PD-L1 and CTLA-4. The function of ICIs and the subsequent receptor inhibition cause hyperactivation of both type 1 T helper lymphocytes (Th1s) and CD8+ cytotoxic T lymphocytes. Notably, preclinical studies in mouse models treated with ICIs have demonstrated an increase in lymphocyte infiltration, and mice with PD-1 receptor knockout show increased inflammatory vascular lesions [51].

ICIs have various methods of action depending on the target:

- The inhibition of CTLA-4 reduces regulatory T lymphocytes (Tregs), which regulate and suppress the immunological response, and stimulates naïve T lymphocytes at the lymph node level, thus boosting their circulation.
- The inhibition of PD-1/PD-L1 recovers the capabilities of exhausted T lymphocytes, enabling the reinstatement of their capacity to generate pro-inflammatory cytokines and, hence, their effector role [52].

The effects of T lymphocyte hyperactivation are expressed intracellularly by means of the activation of signaling channels that allow for the synthesis of cytokines, including the Nuclear Factor of Activated T-Cells (NFAT), Mitogen-Activated Protein Kinase (MAPK), and the nuclear factor kappa-light-chain-enhancer of activated B cells (NF-ΚB) pathways. Among the main cytokines participating in this process is interleukin-6 (IL-6), which directly targets pro-inflammatory conditions and indirectly drives systemic inflammation by stimulating the liver to release acute phase proteins with a resulting pro-thrombotic effect. Interferon gamma (IFN-γ), another significant participant in the process enabling thrombosis, stimulates the immune system and increases the expression of major histocompatibility complex class 1 (MHC I) at the endothelial level, thereby increasing exposure to cytotoxic CD8+ T lymphocytes, promoting endothelial damage, much as Tumor Necrosis Factor Alpha (TNF-α) does, hence amplifying the damage. Furthermore, powering this process is another significant mechanism, known as the feedforward process, activated by chemokines such as C-X-C motif chemokine ligand 9/10 (CXCL9/10), which attract T lymphocytes to the site of inflammation via a positive feedback mechanism. These chemokines are not directly involved in the thrombotic process, but they sustain the inflammatory environment induced by ICI therapy [53,54].

Moreover, ICI-induced T-cell hyperactivation stimulates PD-L1 high monocytes and increases the production of tissue factor (TF), leading to coagulation and fibrinolytic dysregulation [55]. T-cells have thus been shown to play a role in coagulation through cytokine production and direct interactions with endothelial cells, platelets, and other immune components, contributing to a prothrombotic environment [56,57].

### 5.2. Activation of Innate Immunity

Although the molecular data on thrombus formation induced by ICIs is not entirely clear, it is certain that several molecules are involved in their formation. It is a dynamic pathogenic process, in which T lymphocytes and the cytokines they produce are accompanied by components of innate immunity, which in turn promote pro-thrombotic action, thanks in part to a further increase in pro-inflammatory cytokines.

For cancer patients treated with ICIs, MDSCs have been proven to be pro-coagulant agents; they are also suggested to be involved in the process of resistance to immunotherapy. Their immunosuppressive effect is linked with pro-inflammatory activity; in fact, their increase within the tumor mass correlates with a direct increase in the interleukin-8/C-X-C chemokine receptor 1/2 (IL-8/CXCR1/2) axis, demonstrating resistance to certain ICIs such as nivolumab, ipilimumab, and atezolizumab [4,58,59].

The rise in circulating IL-8 activates neutrophils, causing a rise in neutrophil extracellular trap (NET) production. These are fibrous structures containing histones, myeloperoxidase, DNA, and neutrophil elastase with pro-thrombotic action. NETs fulfil two primary functions: they immobilize and eliminate pathogenic microorganisms, and they also provide structural support that promotes the formation of blood clots [60]. Preclinical studies have shown that after treatment with ICIs, thrombus size and neutrophil count increase, which is associated with an increase in spontaneous NET production [61]. This increase directly promotes the mechanism of thrombogenesis through fibrin deposition and platelet activation [62].

Monocytes and macrophages also participate in the immune–thrombotic mechanism activated by ICIs. Their behavior promotes prothrombotic activity on several levels: upregulating endothelial TF expression under lymphocyte stimulation and enhancing platelet aggregation due to increased thrombin production. This action is also based on further rises in IL-6, interleukin-1 (IL-1), and TNF-α [63,64].

### 5.3. Endothelial Dysfunction, Platelet Activation, and Inhibition of Fibrinolysis: The Pro-Thrombotic Role of ICIs

The immune system becomes hyperactive when exposed to ICIs that activate both innate and adaptive immune responses, leading to major vascular equilibrium disturbances, resulting in endothelial dysfunction. The development of immunotherapy-related thrombosis is caused by this imbalance.

The vascular endothelium functions as a selective barrier that maintains equilibrium between coagulation and fibrinolysis processes. The continuous presence of inflammatory cytokines such as IFN-γ, TNF-α and IL-6 in circulation causes endothelial changes, which result in the loss of a protective phenotype and development of a pro-inflammatory and pro-coagulant phenotype [65].

During ICI-associated inflammatory activation, monocytes and neutrophils may release extracellular vesicles containing TF, which are then transmitted to activated platelets. Through the activation of factor VII and the subsequent synthesis of thrombin, a vital component for clot creation, this mechanism starts the coagulation cascade [66].

Endothelial modifications cause overexpression of adhesion molecules on the endothelial surface, including Intercellular Adhesion Molecule 1 (ICAM-1) and Vascular Cell Adhesion Molecule 1 (VCAM-1), which promote the adhesion and transmigration of immune cells [67]. The inflammatory microenvironment also results in a decrease in the production of anticoagulant and fibrinolytic molecules, including thrombomodulin and tissue plasminogen activator (tPA). In addition, plasminogen activator inhibitor-1 (PAI-1) is increased, blocking plasmin activation and thus preventing fibrin degradation and preserving clots.

A key role is also established by the increment in platelets and their increasing activation with cascade amplification. Indeed, platelets, activated by direct contact with damaged endothelium and contact with cells of the immune system and NETs, release pro-coagulant effectors, such as thromboxane A2 and P-selectin. Apart from potentially promoting the activation of TF and factor XII, NETs promote platelet adhesion as well as the adherence of erythrocytes and Von Willebrand Factor (VWF), increasing the thrombotic burden and impairing fibrinolysis [62].

An increase in ATEs, such as stroke, myocardial infarction and peripheral ischemia, has been demonstrated through preclinical studies on murine models and in patients treated with ICIs, a mechanism fueled by the systemic inflammatory state induced by ICIs and by their action, which generates an imbalance in the vascular endothelium [68].

Furthermore, several cases of vasculitis associated with ICIs have been described in the literature. These are rare events but appear to have an important clinical relevance, and from a molecular point of view, they appear to be due to immune hyperactivation, driven above all by the inhibition of PD-1, with a consequent increase in circulating T lymphocytes, which fuel the inflammatory process and cause endothelial damage [69]. Furthermore, an increase in antinuclear antibodies (ANAs) has been found in some patients affected by acral vasculitis associated with ICIs, suggesting an immunological mechanism based on the increase in autoantibodies directed against antigens present both in healthy and pathological tissues, as demonstrated in murine models [70].

Therefore, the different drivers of endothelial dysfunction in ICI-treated patients work together to create a vicious inflammatory–prothrombotic cycle through lymphocyte activation, which establishes a persistent inflammatory microenvironment that activates the endothelium and innate immune cells. The activated endothelium triggers coagulation as platelets consolidate the thrombus, and inhibited fibrinolysis prolongs its duration. The complex combination of factors results in elevated rates of ATEs, VTE and microvascular complications that require close clinical monitoring and individualized therapeutic approaches.

**Figure 1:**
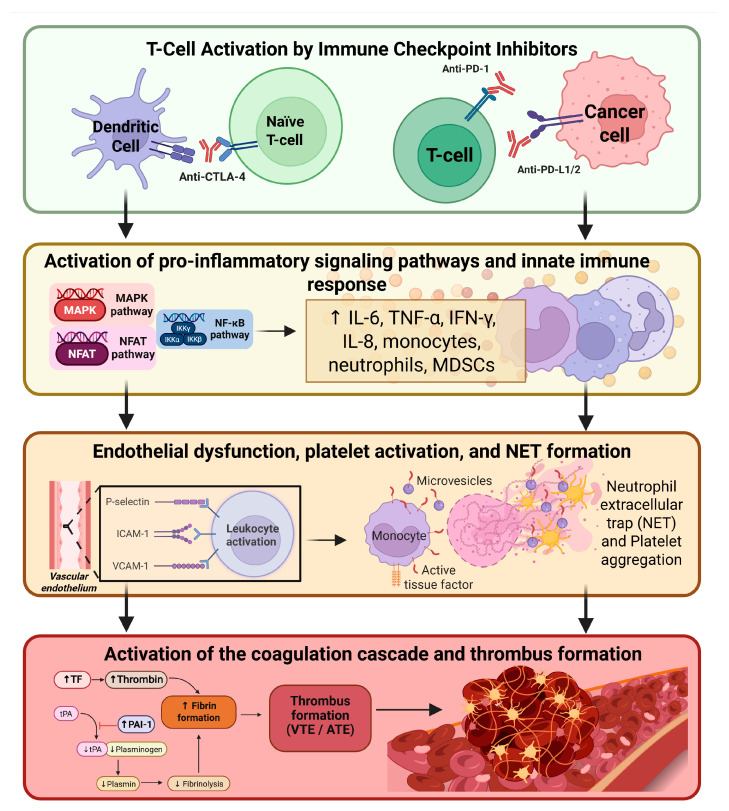
Proposed mechanisms linking immune checkpoint inhibitors (ICIs) to thrombosis. Immune checkpoint inhibitors trigger T-cell activation through PD-1/PD-L1 and CTLA-4 blockade, leading to the engagement of pro-inflammatory intracellular signaling pathways and the release of inflammatory cytokines. The ensuing cytokine-driven activation of innate immune cells promotes endothelial dysfunction, platelet activation and aggregation, NET formation, and TF expression. These vascular and cellular alterations converge upon activation of the coagulation cascade, increased thrombin generation, fibrin deposition, and inhibition of fibrinolysis. Collectively, these processes culminate in the formation of venous and arterial thrombi. Created in BioRender, Ilari, A. (2026) https://BioRender.com/uktf3u6.

Although the data available in the literature allow us to describe biologically plausible mechanisms linking ICIs and thrombosis, this evidence is associative, and further studies are needed to clarify causality. Figure 1 summarizes these proposed mechanisms, which remain hypothetical and not definitive.

## 6. Old and New Predictive Biomarkers

CAT continues to pose a significant medical problem for patients receiving immunotherapy. The risk of blood clots in these patients seems to be affected by more than just the usual factors, such as tumor characteristics, problems with blood vessel lining, or changes in blood coagulation. It also has a connection to the activation of the immune system. This differs from patients who receive chemotherapy. While traditional methods such as the KRS have helped to evaluate the risk of thrombosis in cancer patients, they are not suitable for patients on immunotherapy. Indeed, these models were developed primarily for chemotherapy-related risk profiles; therefore, they fail to capture the immune mechanisms that appear increasingly central to the pathogenesis of CAT during treatment with ICIs [9,24].

The KRS, for example, is based on clinical and laboratory parameters such as tumor type, body mass index and baseline blood values. Although predictive in chemotherapy-treated populations, its accuracy decreases markedly in patients receiving ICI treatment, with sensitivity for VTE often less than 60% [71]. Modified clinical scores such as Vienna CATS and COMPASS-CAT have been developed, which include laboratory biomarkers such as D-dimer and sP-selectin [40,72]. However, these models also fall short, as they are not specifically targeted to the inflammatory and endothelial activation profiles uniquely modulated by immune checkpoint blockade.

### 6.1. Coagulation Biomarkers

D-dimer is one of the most widely used coagulation biomarkers in oncology and has long been associated with an increased risk of VTE in patients with cancer. In patients treated with ICIs, increases in D-dimer levels have been observed in temporal association with thrombotic events; however, there are currently no prospective studies or standardized risk estimates specifically validated in populations treated with ICIs [73].

TF, when carried by extracellular vesicles (EV-TF), helps trigger the extrinsic coagulation pathway. It often rises in patients with advanced cancers. Like D-dimer, high EV-TF levels can predict initial and recurrent thrombotic events [74]. Soluble P-selectin, which shows platelet and endothelial activation, has become an important indicator of clotting risk in cancer patients, as it increases in some patients who develop VTE during immunotherapy [72,75,76].

### 6.2. Immuno-Related Biomarkers

New studies have significantly expanded the landscape of biomarkers associated with thrombosis and immune-related complications, including NETs that help platelets clump together to initiate the coagulation process and lead to fibrin build-up [77].

In the oncology field, increasing evidence is emerging that therapy with ICIs may promote NET formation. This would occur through neutrophil activation mediated by inflammatory cytokines, in particular IFN-γ, produced in abundance following the immune modulation induced by ICIs. This mechanism suggests a possible interaction between enhanced immune response and coagulation activation, with relevant implications for the clinical management of cancer patients treated with immunotherapy [78].

Circulating NET markers—including myeloperoxidase-DNA complexes, citrullinated histone H3 (Cit-H3), and cell-free DNA—are becoming more widely acknowledged as markers of major immune-related adverse events, including systemic autoimmune reactions, in addition to thrombotic risk. These findings support the idea that coagulation activation and immune dysfunction share a single pathophysiological axis, placing NETs as a potential critical node in the balance between therapeutic efficacy and toxicity of ICIs. Looking ahead, monitoring of NET-related biomarkers could represent a useful tool for risk stratification and personalization of therapeutic strategies [79,80].

### 6.3. Inflammatory and Cardiac Biomarkers

In patients undergoing immunotherapy, several inflammatory biomarkers are gaining importance as tools for predicting CAT outcomes. One particularly noteworthy phenomenon is the “CRP flare,” which refers to a sudden increase in C-reactive protein (CRP) levels during the first month of treatment, followed by either a gradual reduction or no change over time. This pattern of temporary inflammatory activation has been associated with a significantly higher risk of VTE, suggesting that monitoring CRP trends could be an early indicator of thrombotic risk in this specific treatment setting. In cohorts of patients receiving ICIs, an early rise in CRP during the first 4–6 weeks of treatment has been linked to an increased risk of VTE, with a reported HR of approximately 2–3; however, clinically validated thresholds are lacking, and prospective confirmation is still required.

A “CRP flare” might also reflect an initial hyperactive immune response triggered by ICIs [81]. Alongside CRP, a sustained increase in circulating pro-inflammatory cytokines like IL-6, IL-8, and interleukin-1 receptor antagonist (IL-1Ra) has been observed in patients who experience thrombotic events during immunotherapy. These molecules play a crucial role in activating vascular endothelium, recruiting neutrophils, and creating a procoagulant environment, thereby strengthening the link between inflammation and coagulation. At the same time, there has been an uptick in soluble vascular adhesion molecules such as VCAM-1 and ICAM-1, which indicate endothelial activation and vascular dysfunction, along with granulocytic-phenotype myeloid suppressor cells (G-MDSCs) that can contribute to thrombosis [82,83].

High-sensitivity cardiac troponins (hs-cTns), biomarkers that are typically used to diagnose myocardial injury, are the subject of another new line of research. It has recently been noted that patients receiving ICIs may be at higher risk of thromboembolic and ischemic events if they have even small subclinical increases in troponins. This implies that ICIs may indirectly contribute to the pathophysiology of thrombosis by promoting wider systemic endothelial or microvascular dysfunction in addition to the direct cardiotoxic risk. Therefore, adding troponins to a multimodal panel of biomarkers may be beneficial for early cardiovascular and thrombotic risk stratification [81,84].

Overall, these data reinforce the importance of dynamic and multidimensional monitoring of inflammatory, immune, and vascular biomarkers in cancer patients undergoing immunological therapy with the aim of promptly identifying subjects at risk and preventing the development of potentially severe thrombotic complications.

### 6.4. Tumor-Intrinsic and Genomic Biomarkers

In addition to the role of circulating markers, factors intrinsic to tumors and their genomic characteristics are playing an increasingly important role in understanding the thrombotic risk faced by cancer patients undergoing immunotherapy. A prime example of this is podoplanin, a transmembrane glycoprotein from the mucin family that is often overexpressed in aggressive tumors like glioblastoma, squamous cell lung carcinoma, and certain testicular cancers. By binding to the CLEC-2 receptor found on platelets, podoplanin triggers platelet activation and thrombus formation, especially in the central nervous system. Research, including preclinical and observational studies, has revealed a strong link between elevated podoplanin levels in tumors and the occurrence of cerebral and disseminated venous thrombosis [85,86].

At the same time, emerging genomic data is shedding light on the significant role that specific somatic mutations play in creating a prothrombotic environment. In addition to providing a growth advantage to the tumor, inactivating mutations in genes like *STK11 (LKB1)*, *KEAP1*, and *TP53* alter the tumor microenvironment, fostering oxidative stress, inflammation, and the expression of procoagulant genes. Notably, a 3-fold increase in the incidence of CAT has been observed with the advent of ICIs, particularly linked to co-mutations in *KEAP1* and *STK11* in NSCLC, with a risk as high as 30% within the first six months of treatment [87,88,89].

These results demonstrate how signals from tumors and genetic alterations can interoperate to set off molecular circuits that encourage thrombogenesis, in part by changing the phenotype of endothelial cells and producing sterile vascular inflammation. This data makes it possible to develop thorough and customized thrombotic risk prediction models to guide immunotherapy and preventative and treatment choices for patients.

### 6.5. Emerging Molecular Biomarkers

Translational research is leading to the development of a new class of molecular biomarkers that provide patients with solid tumors with a more dynamic and individualized understanding of their thrombotic risk. Two biomarkers show promise as research tools: platelet RNA and circulating tumor DNA (ctDNA).

ctDNA consists of DNA fragments released into the bloodstream by apoptotic or necrotic tumor cells. Its presence and dynamic changes during treatment reflect tumor mass, cell turnover, and genomic instability, all of which are implicated in promoting a pro-thrombotic environment. Through the examination of ctDNA mutations, changes in genes like *TP53* and *KRAS* have been found to result in elevated TF expression, which raises the risk of thrombosis by triggering the extrinsic coagulation pathway [90,91]. Studies on cell lines and tissues from patients with colorectal cancer and NSCLC have shown that the presence of *KRAS/TP53* mutations correlates with elevated TF levels, suggesting a direct connection between tumor genomic instability and activation of the coagulation cascade [92]. The implementation of ctDNA in disease monitoring may, therefore, offer an early and dynamic indicator of endothelial stress and thrombotic activation [93].

Platelet RNA profiling also opens new perspectives to understanding tumor-host interactions. Platelets internalize exogenous RNA from tumor and immune microvesicles, modifying their transcriptome and influencing functions, such as adhesion, immune signaling, inflammation, and platelet aggregation [94,95].

At present, ctDNA and platelet RNA have the potential to serve as highly sensitive and temporally dynamic biomarkers of thrombo-inflammatory risk, although this is still in the exploratory stage. The prediction of thrombotic risk in cancer patients may be completely transformed by the incorporation of these biomarkers into machine learning-based multi-omics predictive algorithms, thus opening the door to tailored prevention, particularly for patients receiving ICI treatment.

At the same time, new data suggest that metabolic remodeling associated with the pro-coagulant phenotype may also offer useful predictive models. Certain metabolites, such as pyruvate, acetate, glycine, glutamine, valine, leucine, and isoleucine, have been identified as potential biomarkers associated with thrombotic risk during treatment with ICIs. Monitoring metabolic profiles during follow-up could be an effective strategy for anticipating the onset of cancer-associated thromboembolic events, enabling timely and personalized clinical interventions [96].

Metabolic markers as well as platelet RNA are host-related, as are the coagulation-, immune-, inflammation-, and cardiac-related molecules described above (Table 2). Conversely, somatic mutations, podoplanin, and ctDNA are closely related to the tumor (Table 3).

### 6.6. Clinical Implications of Predictive Biomarkers in ICI-Associated CAT

Biomarkers associated with thrombotic risk in cancer patients treated with ICIs play a controversial role in clinical practice, and their use depends on several factors, including the reproducibility of dosages and availability in outpatient settings. For these reasons, it is possible to distinguish between clinically usable biomarkers and exploratory biomarkers.

D-dimer and CRP are the most promising candidates for clinical translation, thanks to their wide availability and established use in oncology. In patients treated with ICIs, these markers may show dynamic changes after the start of therapy, suggesting a possible link between immune activation and thrombotic risk. Conversely, biomarkers related to NETosis or extracellular vesicles are currently difficult to apply in everyday clinical practice due to the absence of standardized methods and validated threshold values.

Therefore, predictive biomarkers should be considered complementary tools to the clinical models currently used to stratify patients at risk of VTE, pending studies that can validate their use in clinical practice. A summary of the main available evidence is shown in Supplementary Table S1.

## 7. Discussion

Over the past two decades, the advent of immunotherapy has profoundly transformed the therapeutic landscape of several malignancies. ICIs were hypothesized to be associated with an increased risk of thrombosis, given the close interplay between immune activation, inflammation, and CAT [97,98]. However, due to the limited amount of available evidence, the true incidence of CAT in patients receiving ICIs, the impact of these events on survival outcomes, and the identification of specific risk factors remain largely undefined.

Accurate risk stratification and effective prevention of CAT are, therefore, of paramount importance. As previously discussed, the cornerstone of prevention lies in the identification of patients at high thrombotic risk based on individual clinical characteristics and established risk factors [24,31,36,99,100].

With regard to currently available RAMs, it should be emphasized that all were developed in the pre-immunotherapy era. Consequently, the identification of ICI-specific risk factors and biomarkers for predicting CAT—an essential prerequisite for modern clinical practice—remains an unmet need.

Moreover, existing RAMs provide only a static and general estimation of thrombotic risk, failing to capture the dynamic and patient-specific evolution of disease and treatment, including changes in blood values, tumor progression [101], hospitalization [102], invasive procedures [103], and changes in therapeutic regimens [104].

Regarding CAT prevention, primary anticoagulant prophylaxis in the outpatient setting is still infrequently implemented in routine clinical practice. Current international guidelines recommend thromboprophylaxis with either low-molecular-weight heparin (LMWH) or direct oral anticoagulants (DOACs) for ambulatory patients at intermediate-to-high risk of VTE, as assessed by validated risk scores [27,105].

The meta-analysis conducted by Bosch et al. confirmed the protective effect of primary thromboprophylaxis with both LMWH and DOACs, demonstrating an approximately 50% reduction in thrombotic events among patients with cancer [106].

Notably, the TARGET-TP study adopted a biomarker-driven risk stratification approach based on D-dimer and fibrinogen levels. This study demonstrated that primary thromboprophylaxis significantly reduced the incidence of thrombosis in the high-risk group compared with the low-risk group. Although limited by a relatively small sample size (*n* = 328), a reduction in mortality associated with thromboprophylaxis was also observed [107].

Nevertheless, further studies with larger cohorts are required to validate these findings. In particular, the effect of primary thromboprophylaxis on overall survival remains uncertain, as current evidence from clinical trials is inconclusive [108,109].

The management and prevention of CAT primarily involve the use of LMWHs and DOACs. As these agents demonstrate comparable efficacy and safety profiles, treatment selection should be individualized based on thrombotic and bleeding risk, potential drug–drug interactions, polypharmacy, and patient preferences. Indeed, in patients with lung cancer, polypharmacy is particularly common due to advanced age, comorbidities, and frailty. The prevalence of drug–drug interactions increases from approximately 14% in patients receiving up to four medications to 67% in those taking more than eleven drugs [110].

Furthermore, all DOACs are substrates of P-glycoprotein and cytochrome P450 enzymes; therefore, concomitant therapies that inhibit or induce these pathways may significantly alter DOAC pharmacokinetics [111,112]. Apixaban, edoxaban, and rivaroxaban are substrates of the cytochrome P450 3A4 (CYP3A4) enzyme system.

Consequently, clinicians should carefully evaluate the potential for drug–drug interactions when selecting anticoagulant therapy for CAT in patients treated with some oncology drugs such as tyrosine kinase inhibitors, docetaxel, paclitaxel, irinotecan, and vinca alkaloids. In the context of extensive polypharmacy or altered pharmacokinetics (e.g., renal impairment or obesity), LMWHs may represent a safer option due to their lower interaction potential [27].

The recommended duration of anticoagulant therapy is generally 3–6 months and typically continues as long as the cancer remains active. According to current definitions, cancer is considered active in the presence of metastatic disease and/or ongoing antineoplastic treatment, whereas remission lasting at least six months is regarded as inactive disease. Evidence indicates that premature discontinuation of anticoagulation while cancer remains active is associated with a more than six-fold increase in VTE recurrence [113,114].

Rather than complete treatment cessation, dose reduction may represent a safer alternative. Recently, a large randomized controlled trial demonstrated that extended anticoagulation with reduced-dose apixaban (2.5 mg twice daily) was non-inferior to full-dose apixaban for preventing recurrent VTE in patients with active cancer, significantly reducing the incidence of clinically relevant bleeding events [115].

Furthermore, low-intensity therapy with apixaban 2.5 mg twice daily for 12 months showed a lower risk of symptomatic recurrent VTE than placebo, with a low risk of major bleeding [116].

Despite these advances, optimal anticoagulant choice and treatment duration in patients with active cancer remain areas requiring further investigation, particularly in the era of prolonged survival and long-term systemic therapies.

However, patients who complete treatment with immunotherapy and have no evidence of active disease, either in an adjuvant or advanced setting, may experience long-term survival and, therefore, be suitable for clinical monitoring without anticoagulation.

The routine integration of biomarkers into clinical practice warrants further validation; however, several biomarkers appear promising for monitoring and preventing CAT. Hemostatic biomarkers such as D-dimer and sP-selectin are associated with hypercoagulability, increased VTE risk, and poor prognosis in patients with cancer [72,117]. In the Vienna CAT-BLED study, sP-selectin emerged as a strong predictive biomarker and demonstrated a correlation with D-dimer levels, supporting the hypothesis that platelet activation occurs in parallel with enhanced procoagulant and fibrinolytic activity, ultimately reflecting tumor progression or increased tumor burden [118].

Therefore, serial monitoring of D-dimer and/or sP-selectin may provide a dynamic tool for assessing evolving thromboembolic risk by capturing changes in the patient’s pro-coagulatory state over time.

Moreover, tumor-intrinsic biomarkers such as *STK11*, *KEAP11*, and *TP53* in NSCLC may guide the decision to administer prophylaxis to these patients, especially those who receive immunotherapy–chemotherapy combinations.

Recently, the Vienna CATS score was externally validated in a prospective cohort of patients receiving contemporary cancer treatments, including immunotherapy, across multiple longitudinal time points. The study confirmed good predictive accuracy for 6-month VTE risk assessment and demonstrated the dynamic applicability of this model in clinical practice [39].

Finally, the potential link between ICI-induced inflammation and hypercoagulability has prompted investigation into the role of CRP as a predictor of VTE risk. Emerging data in patients treated with ICIs indicate a correlation between CRP fluctuations and ICI-associated VTE [82]. If confirmed in future studies, early increases in CRP levels could serve as a practical and accessible biomarker to identify patients at high risk of thromboembolic events who may benefit from thromboprophylaxis after ruling out active thrombosis.

In summary, the role of biomarkers that may predict thrombosis in patients treated with ICIs remains controversial. Initial changes in biomarkers observed during treatment with ICIs may represent transient phenomena linked exclusively to immune activation, rather than the expression of an established thrombotic risk, making the use of fixed thresholds in clinical decisions inappropriate. Consequently, the longitudinal interpretation of biomarkers should be aimed primarily at clinical monitoring and dynamic risk stratification, pending the necessary and lacking prospective evidence that would allow their use in thromboprophylaxis. However, the integration of the available validated RAMs, such as the KRS and the Vienna CATS score, with new potential biomarkers (D-dimer, sP-selectin, and CRP) could help clinicians to identify high-risk patients who may benefit from thromboprophylaxis.

**Figure 2:**
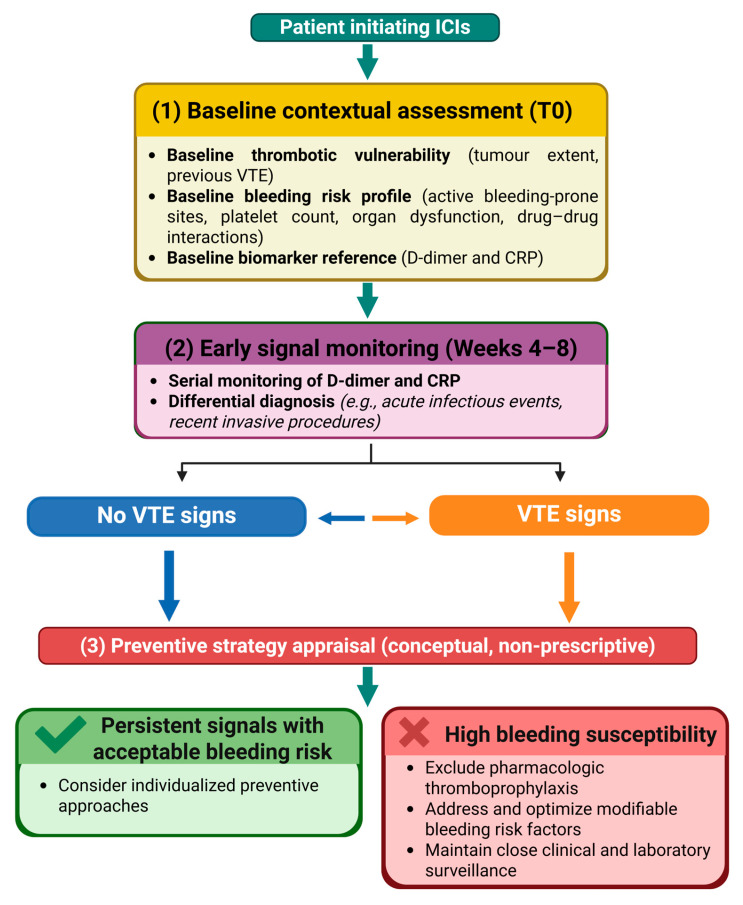
Conceptual framework for contextual risk assessment and biomarker monitoring in patients initiating ICIs. The diagram illustrates a stepwise approach, including baseline evaluation of thrombotic vulnerability, bleeding risk, and biomarker reference values, followed by early monitoring of D-dimer and CRP with clinical contextualization. The framework is intended to support hypothesis generation and risk awareness rather than to provide prescriptive guidance for thromboprophylaxis.

Given the limitations of current risk models and biomarker evidence in the immunotherapy setting, we propose a conceptual, hypothesis-generating framework to support baseline assessment and early biomarker monitoring in patients receiving ICIs (Figure 2). This model is not intended for clinical decision-making but to provide a biological and clinical context for future prospective validation.

## 8. Conclusions

Patients with cancer have a high risk of CAT, which affects their quality of life, often resulting in high rates of hospitalization and mortality, especially in the first year from diagnosis.

An increase in thrombotic phenomena, both VTE and ATEs, has been demonstrated not only in patients treated with chemotherapy but also in patients treated with immunotherapy, especially when used in combination. Despite the widespread use of RAMs for stratifying risk and evaluating thromboprophylaxis in patients undergoing chemotherapy, the assessment of the risk of thromboembolic events and its prevention in patients treated with ICIs is dictated more by clinical and individual experience than by predictive and validated RAMs.

Although no specific recommendations are currently available for assessing specifically the risk of venous and arterial thromboembolic events in patients treated with immunotherapy or immunotherapy–chemotherapy combinations, it is important to remind clinicians that these events may be associated with this class of agents.

However, new promising predictive biomarkers are emerging. Among them, increased levels of D-dimer, sP-selectin, extracellular vesicles containing tissue factor, and circulating cytokines such as IL-6 and IL-8 have been identified as biomarkers useful for stratifying thrombotic risk in patients. Furthermore, at the genomic level, either the presence of mutations in *STK11*, *KEAP1*, and *TP53* or the overexpression of podoplanin may contribute to increasing thrombotic risk. The integration of traditional clinical models with molecular biomarkers and genomic information could enable the development of predictive models to encourage the early diagnosis of CAT and implement prophylactic strategies in patients treated with ICIs.

In this context, the PRIN project (NCT07288632) is currently underway, with the aim of filling the gap in the literature on studies demonstrating a cause–effect correlation between the action of ICIs and the subsequent development of thrombosis. This is a prospective, multicenter observational study evaluating the correlation between genomic profile and thromboembolic risk in patients with metastatic NSCLC undergoing first-line treatment and biomonitoring via CPR, D-dimer and coagulation profile (PT and PTT) testing. Specifically, the primary outcome is to determine the levels of plasma TF, thrombin generation, IL6, vWF, ADAMTS-13 activity, PAI-1, and sP-selectin in patients with advanced or metastatic NSCLC before starting the new line of anticancer therapy and after 3 and 6 months of anticancer treatment, across different patterns and association of oncogene mutations. The study lays the foundation for the construction of specific predictive models for patients treated with ICIs.

Therefore, more studies are needed to understand the underlying mechanisms between CAT and ICIs and to assess the VTE risk in cancer patients undergoing immunotherapy. Finally, specific RAMs should be developed and investigated in clinical trials. These models should be multiparametric and able to evaluate multiple aspects, such as clinical, molecular and genomic components, to increase the predictability of thrombotic risk in patients treated with ICIs.

## Figures and Tables

**Table 1:** **Main characteristics and features of RAMs used in CAT.**

RAM	Cancer Type	Parameters
CATSCORE	Breast, Prostate, Lung, Esophagus, Gastrointestinal, Kidney, Lymphoma, Urothelial, Endometrial, Ovarian, Pancreas, Stomach	Site of cancer, D-dimer
COMPASS-CAT	Breast, Colorectal, Lung and Ovarian	Anthracycline or anti-hormonal therapy, time since cancer diagnosis, central venous catheter, stage of cancer, presence of CV risk factors, recent hospitalization for acute medical illness, personal history of VTE, PLT count ≥ 350.000
Khorana Risk Score (KRS)	Lung, Colorectal, Breast, Ovarian, Sarcoma and Lymphomas	Hb ≤ 10 g/dL and/or use of ESA, site of cancer, PLT count ≥ 350.000/L, WBC count ≥ 11.000/L, BMI ≥ 35 kg/m^2^
MDACC	Breast, Gastrointestinal, Pancreas, Urothelial, Kidney, Gynecological, Lung and Lymphoma	Presence of metastasis, use of platinum-based chemotherapy, use of ESAs, site of cancer
ONKOTEV	Any	KRS > 2, personal history of VTE, metastatic disease, vascular/lymphatic macroscopic compression
PROTECHT	Gastrointestinal, Breast, Ovarian, Lung, Pancreas, Head and Neck	Adding carboplatin, gemcitabine and/or cisplatin to KRS parameters
Tic-ONCO	Not Available	Adding genetic risk score to KRS
Vienna-CATS	Breast, Lung, Gastrointestinal, Pancreas, Kidney, Prostate, Brain, Lymphoma and Multiple Myeloma	Adding D-dimer and P-selectin levels to KRS

CV, cardiovascular; ESA, erythropoietin-stimulating agent; KRS, Khorana risk score; Hb, hemoglobin; WBC, white blood cell; PLT, platelet; VTE, venous thromboembolism; BMI, body mass index.

**Table 2:** **Extended table of host-derived biomarkers associated with thrombosis in ICI therapy.** This table describes some of the main markers related to thrombosis and ICIs. These are molecules directly and indirectly involved in the thrombotic and immunological mechanisms and are closely related to the host.

Category	Biomarker	Function/Mechanism	Clinical Significance	Relevance with ICIs
**Coagulation**	D-dimer	Fibrin degradation product	Associated with elevated risk of VTE	Post-ICI elevation → HR > 2
	EV-TF	Helps start the extrinsic coagulation pathway	Predictive of initial and recurrent thrombosis	↑ in advanced cancers
	P-selectin	Platelet/endothelial activation marker	Sign of clots in cancer patients	↑ in patients under ICIs
**Immuno-related**	NETs	Neutrophil extracellular traps	Trigger the mechanism of thrombosis and platelet accumulation	ICIs are linked to NETosis
	MPO-DNA, Cit-H3, cfDNA	Circulating NET markers	Increase Immune-related adverse events and VPE risk	Emerging risk stratification tools
**Inflammation**	CRP	Acute phase protein	Early indicator of thrombotic risk	Sign of immune activation under ICIs
	IL-6, IL-8, IL-1Ra	Pro-inflammatory cytokines	Promote procoagulant microenvironment	↑ in patients under ICI-associated with VTE
	VCAM-1, ICAM-1	Endothelial adhesion molecules	Markers of endothelial activation and vascular dysfunction	Expression increased by ICIs
	G-MDSC	Immunosuppressive cells	Contribute to thrombosis via inflammation	↑ during ICI therapy
**Cardiac**	hs-cTn	Myocardial injury marker	Linked to TE and ischemic events	Indicating endothelial and microvascular dysfunction by ICIs
**Molecular**	Platelet RNA	Tumor RNA internalized by platelets	Modifies platelet transcriptome	Emerging CAT prediction marker
**Metabolic**	Metabolic profile (e.g., pyruvate, glycine…)	Procoagulant metabolic remodeling	Potential predictor of CAT risk	Dynamic monitoring during ICI treatment

**Table 3:** **Extended table of tumor-derived biomarkers associated with thrombosis in ICI therapy.** This table describes the markers that are correlated with the tumor at multiple levels: genomic, intrinsic, and molecular. These factors also vary according to tumor type.

Genomic	*STK11*, *KEAP1, TP53*	Pro-Inflammatory Somatic Mutations	Create Prothrombotic Tumor Microenvironment	↑ CAT Risk with Co-Mutations in *KEAP1* and *STK11*
**Tumor-intrinsic**	Podoplanin	Binding the CLEC-2 receptor -> platelet activation and thrombus formation	Associated with cerebral and disseminated VPE thrombosis	Overexpressed in aggressive tumors (NSCLC, GBM, testicular tumor)
**Molecular**	ctDNA	DNA fragments released into the bloodstream by apoptotic or necrotic tumor cells	Reflects tumor burden, TF activation	*TP53/KRAS* mutations → ↑ thrombosis risk

## Data Availability

No new data were created or analyzed in this study. Data sharing is not applicable to this article.

## Supplementary Materials

The following supporting information can be downloaded at https://www.mdpi.com/article/10.3390/ijms27041874/s1.

## Abbreviations

The following abbreviations are used in this manuscript:

ATE	Arterial Thromboembolic Event
CAT	Cancer-Associated Thrombosis
CATS	Cancer and Thrombosis Study
cfDNA	Cell-Free DNA
ctDNA	Circulating Tumor DNA
Cit-H3	Citrullinated Histone H3
COMPASS-CAT	Comparison of Methods for Thromboembolic Risk Assessment with Clinical Perceptions and AwareneSS in Cancer-Associated Thrombosis
CRP	C-Reactive Protein
CTLA-4	Cytotoxic T-Lymphocyte–Associated Protein 4
CXCL	C-X-C Motif Chemokine Ligand
CXCR	C-X-C Chemokine Receptor
DIC	Disseminated Intravascular Coagulation
DOACs	Direct Oral Anticoagulants
EV-TF	Extracellular Vesicle-Associated Tissue Factor
G-MDSC	Granulocytic Myeloid-Derived Suppressor Cell
hs-cTn	High-Sensitivity Cardiac Troponin
ICAM-1	Intercellular Adhesion Molecule 1
ICIs	Immune Checkpoint Inhibitors
IFN-γ	Interferon Gamma
IL	Interleukin
IL-1Ra	Interleukin 1 Receptor Antagonist
irAEs	Immune-Related Adverse Events
KRS	Khorana Risk Score
LL-DVT	Lower-Limb Deep Vein Thrombosis
MAPK	Mitogen-Activated Protein Kinase
MDACC-CAT	MD Anderson Cancer Center Cancer-Associated Thrombosis Model
MDSCs	Myeloid-Derived Suppressor Cells
MHC I	Major Histocompatibility Complex Class I
NETs	Neutrophil Extracellular Traps
NF-KB	Nuclear Factor Kappa-Light-Chain-Enhancer of Activated B Cells
NFAT	Nuclear Factor of Activated T-cells
NSCLC	Non-Small Cell Lung Cancer
OAC	Oral Anticoagulant
OR	Odds Ratio
PAI-1	Plasminogen Activator Inhibitor 1
PD-1	Programmed Death 1
PD-L1	Programmed Death-Ligand 1
PE	Pulmonary Embolism
PLT	Platelets
RAMs	Risk Assessment Models
sP-selectin	Soluble P-selectin
TF	Tissue Factor
TNF-α	Tumor Necrosis Factor Alpha
UE-DVT	Upper-Extremity Deep Vein Thrombosis
VCAM-1	Vascular Cell Adhesion Molecule 1
VTE	Venous Thromboembolism
VWF	Von Willebrand Factor
WBC	White Blood Cell

## References

[1] Bailey A.J.M., Luo O.D., Zhou S.Q., Wells P.S. (2025). The incidence and risk of venous thromboembolism in patients with active malignancy and isolated superficial venous thrombosis: A systematic review and meta-analysis (IROVAM–iSVT review). J. Thromb. Haemost..

[2] Khorana A.A., Francis C.W., Culakova E., Kuderer N.M., Lyman G.H. (2007). Thromboembolism is a leading cause of death in cancer patients receiving outpatient chemotherapy. J. Thromb. Haemost..

[3] Solinas C., Saba L., Sganzerla P., Petrelli F. (2020). Venous and arterial thromboembolic events with immune checkpoint inhibitors: A systematic review. Thromb. Res..

[4] Roopkumar J., Swaidani S., Kim A.S., Thapa B., Gervaso L., Hobbs B.P., Wei W., Alban T.J., Funchain P., Kundu S. (2021). Increased Incidence of Venous Thromboembolism with Cancer Immunotherapy. Med.

[5] Moik F., Chan W.S.E., Wiedemann S., Hoeller C., Tuchmann F., Aretin M.B., Fuereder T., Zöchbauer-Müller S., Preusser M., Pabinger I. (2021). Incidence, risk factors, and outcomes of venous and arterial thromboembolism in immune checkpoint inhibitor therapy. Blood.

[6] Sussman T.A., Li H., Hobbs B., Funchain P., McCrae K.R., Khorana A.A. (2021). Incidence of thromboembolism in patients with melanoma on immune checkpoint inhibitor therapy and its adverse association with survival. J. Immunother. Cancer.

[7] De Azevedo L.A., Orione C., Tromeur C., Couturaud F., Descourt R., Geier M. (2024). Incidence of venous thromboembolism and association with PD-L1 expression in advanced non-small cell lung cancer patients treated with first-line chemo-immunotherapy. Front. Oncol..

[8] Peng Q., Zhu J., Zhang Y., Jing Y. (2024). Blood hypercoagulability and thrombosis mechanisms in cancer patients -A brief review. Heliyon.

[9] Mulder F.I., Horváth-Puhó E., van Es N., van Laarhoven H.W.M., Pedersen L., Moik F., Ay C., Büller H.R., Sørensen H.T. (2021). Venous thromboembolism in cancer patients: A population-based cohort study. Blood.

[10] Verso M., Giustozzi M., Vinci A., Franco L., Vedovati M.C., Marchesini E., Becattini C., Agnelli G. (2021). Risk factors and one-year mortality in patients with direct oral anticoagulant-associated gastrointestinal bleeding. Thromb. Res..

[11] Verso M., Agnelli G. (2020). Treatment of venous thromboembolism in patients with cancer: From clinical trials to real life. Thromb. Res..

[12] Blom J.W., Doggen C.J.M., Osanto S., Rosendaal F.R. (2005). Malignancies, prothrombotic mutations, and the risk of venous thrombosis. JAMA.

[13] Fernandes C.J., Morinaga L.T.K., Alves J.L., Castro M.A., Calderaro D., Jardim C.V.P., Souza R. (2019). Cancer-associated thrombosis: The when, how and why. Eur. Respir. Rev..

[14] Ma Z., Sun X., Zhang Y., Li H., Sun D., An Z., Zhang Y. (2022). Risk of Thromboembolic Events in Cancer Patients Treated with Immune Checkpoint Inhibitors: A Meta-analysis of Randomized Controlled Trials. Thromb. Haemost..

[15] Moik F., Horváth-Puhó E., Ay C., Pabinger I., Mulder F., van Es N., Sørensen H.T. (2025). Arterial and venous thromboembolic events in patients with cancer treated with targeted therapies: A population-based cohort study. eClinicalMedicine.

[16] Kewan T., Ko T., Flores M., Sallam Y., Haddad A., Daw H. (2021). Prognostic impact and risk factors of cancer-associated thrombosis events in stage-IV cancer patients treated with immune checkpoint inhibitors. Eur. J. Haematol..

[17] Sheng I.Y., Gupta S., Reddy C.A., Angelini D., Funchain P., Sussman T.A., Sleiman J., Ornstein M.C., McCrae K., Khorana A.A. (2022). Thromboembolism in Patients with Metastatic Urothelial Cancer Treated with Immune Checkpoint Inhibitors. Target Oncol..

[18] Li A., May S.B., La J., Martens K.L., Amos C.I., Flowers C.R., Do N.V., Brophy M.T., Chitalia V., Ravid K. (2023). Venous thromboembolism risk in cancer patients receiving first-line immune checkpoint inhibitor versus chemotherapy. Am. J. Hematol..

[19] Khorana A.A., Palaia J., Rosenblatt L., Pisupati R., Huang N., Nguyen C., Barron J., Gallagher K., Bond T.C. (2023). Venous thromboembolism incidence and risk factors associated with immune checkpoint inhibitors among patients with advanced non-small cell lung cancer. J. Immunother. Cancer.

[20] Deschênes-Simard X., Richard C., Galland L., Blais F., Desilets A., Malo J., Cvetkovic L., Belkaid W., Elkrief A., Gagné A. (2021). Venous thrombotic events in patients treated with immune checkpoint inhibitors for non-small cell lung cancer: A retrospective multicentric cohort study. Thromb. Res..

[21] Doubre H., Greillier L., Justeau G., Ricordel C., Swalduz A., Curcio H., Bylicki O., Auliac J.B., Guisier F., Bigay-Game L. (2023). Venous thrombotic events and impact on outcomes in patients treated with first-line single-agent pembrolizumab in PD-L1 ≥ 50% advanced non small cell lung cancer. J. Cancer Res. Clin. Oncol..

[22] Lin J., Li W., Zhang X., Zhou K., Yang Y., Cheng S., Sun R., Dang C., Diao D. (2025). Thromboembolic events associated with immune checkpoint inhibitors in cancer patients: A Bayesian network meta-analysis. Thromb. Res..

[23] Khorana A.A., Francis C.W. (2018). Risk prediction of cancer-associated thrombosis: Appraising the first decade and developing the future. Thromb. Res..

[24] Khorana A.A., Kuderer N.M., Culakova E., Lyman G.H., Francis C.W. (2008). Development and validation of a predictive model for chemotherapy- associated thrombosis. Blood.

[25] Streiff M.B., Holmstrom B., Angelini D., Ashrani A., Buckner T., Diep R., Fertrin K.Y., Fogerty A.E., Gallastegui Crestani N., Gangaraju R. (2024). Cancer-Associated Venous Thromboembolic Disease, Version 2.2024, NCCN Clinical Practice Guidelines in Oncology. J. Natl. Compr. Cancer Netw..

[26] https://www.iss.it/documents/20126/8403839/LG_227_TEV_Tumori_Solidi_agg2024.pdf/35d01dbf-7c52-7c94-110d-6c561ecd82f7?t=1715843145115.

[27] Falanga A., Ay C., Di Nisio M., Gerotziafas G., Jara-Palomares L., Langer F., Lecumberri R., Mandala M., Maraveyas A., Pabinger I. (2023). Venous thromboembolism in cancer patients: ESMO Clinical Practice Guideline. Ann. Oncol..

[28] Key N.S., Khorana A.A., Kuderer N.M., Bohlke K., Lee A.Y.Y., Arcelus J.I., Wong S.L., Balaban E.P., Flowers C.R., Gates L.E. (2023). Venous Thromboembolism Prophylaxis and Treatment in Patients With Cancer: ASCO Guideline Update. J. Clin. Oncol..

[29] Mulder F.I., Candeloro M., Kamphuisen P.W., Di Nisio M., Bossuyt P.M., Guman N., Smit K., Büller H.R., van Es N., CAT-prediction collaborators (2019). The Khorana score for prediction of venous thromboembolism in cancer patients: A systematic review and meta-analysis. Haematologica.

[30] Ay C., Dunkler D., Marosi C., Chiriac A.L., Vormittag R., Simanek R., Quehenberger P., Zielinski C., Pabinger I. (2010). Prediction of venous thromboembolism in cancer patients. Blood.

[31] Englisch C., Nopp S., Moik F., Starzer A.M., Quehenberger P., Preusser M., Berghoff A.S., Ay C., Pabinger I. (2025). The Vienna CATScore for predicting cancer-associated venous thromboembolism: An external validation across multiple time points. ESMO Open.

[32] Pabinger I., van Es N., Heinze G., Posch F., Riedl J., Reitter E.M., Di Nisio M., Cesarman-Maus G., Kraaijpoel N., Zielinski C.C. (2018). A clinical prediction model for cancer-associated venous thromboembolism: A development and validation study in two independent prospective cohorts. Lancet Haematol..

[33] Verzeroli C., Giaccherini C., Russo L., Bolognini S., Gamba S., Tartari C.J., Schieppati F., Ticozzi C., Vignoli A., Masci G. (2023). Utility of the Khorana and the new-Vienna CATS prediction scores in cancer patients of the HYPERCAN cohort. J. Thromb. Haemost..

[34] Bevan S., Longstaff C. (2022). Is it possible to make a common reference standard for D-dimer measurements? Communication from the ISTH SSC subcommittee on fibrinolysis. J. Thromb. Haemost..

[35] Barni S., Labianca R., Agnelli G., Bonizzoni E., Verso M., Mandalà M., Brighenti M., Petrelli F., Bianchini C., Perrone T. (2011). Chemotherapy-associated thromboembolic risk in cancer outpatients and effect of nadroparin thromboprophylaxis: Results of a retrospective analysis of the PROTECHT study. J. Transl. Med..

[36] Verso M., Agnelli G., Barni S., Gasparini G., Labianca R. (2012). A modified Khorana risk assessment score for venous thromboembolism in cancer patients receiving chemotherapy: The Protecht score. Intern. Emerg. Med..

[37] van Es N., Di Nisio M., Cesarman G., Kleinjan A., Otten H.M., Mahé I., Wilts I.T., Twint D.C., Porreca E., Arrieta O. (2017). Comparison of risk prediction scores for venous thromboembolism in cancer patients: A prospective cohort study. Haematologica.

[38] Di Nisio M., van Es N., Rotunno L., Anzoletti N., Falcone L., De Tursi M., Natoli C., Tinari N., Cavallo I., Valeriani E. (2019). Long-term performance of risk scores for venous thromboembolism in ambulatory cancer patients. J. Thromb. Thrombolysis.

[39] Cella C.A., Di Minno G., Carlomagno C., Arcopinto M., Cerbone A.M., Matano E., Tufano A., Lordick F., De Simone B., Muehlberg K.S. (2017). Preventing venous thromboembolism in ambulatory cancer patients: The ONKOTEV Study. Oncologist.

[40] Gerotziafas G.T., Taher A., Abdel-Razeq H., AboElnazar E., Spyropoulos A.C., El Shemmari S., Larsen A.K., Elalamy I., COMPASS–CAT Working Group (2017). A Predictive Score for Thrombosis Associated with Breast, Colorectal, Lung, or Ovarian Cancer: The Prospective COMPASS-Cancer-Associated Thrombosis Study. Oncologist.

[41] Spyropoulos A.C., Eldredge J.B., Anand L.N., Zhang M., Qiu M., Nourabadi S., Rosenberg D.J. (2020). External Validation of a Venous Thromboembolic Risk Score for Cancer Outpatients with Solid Tumors: The COMPASS-CAT Venous Thromboembolism Risk Assessment Model. Oncologist.

[42] Rupa-Matysek J., Lembicz M., Rogowska E.K., Gil L., Komarnicki M., Batura-Gabryel H. (2018). Evaluation of risk factors and assessment models for predicting venous thromboembolism in lung cancer patients. Med. Oncol..

[43] Rojas-Hernandez C.M., Tang V.K., Sanchez-Petitto G., Qiao W., Richardson M., Escalante C. (2020). Development of a clinical prediction tool for cancer-associated venous thromboembolism (CAT): The MD Anderson Cancer Center CAT model. Support. Care Cancer.

[44] Muñoz Martín A.J., Ortega I., Font C., Pachón V., Castellón V., Martínez-Marín V., Salgado M., Martínez E., Calzas J., Rupérez A. (2018). Multivariable clinical-genetic risk model for predicting venous thromboembolic events in patients with cancer. Br. J. Cancer.

[45] Icht O., Darzi N., Shimony S., Jacobi O., Reinhorn D., Landman Y., Mutai R., Averbuch I., Shochat T., Spectre G. (2021). Venous thromboembolism incidence and risk assessment in lung cancer patients treated with immune checkpoint inhibitors. J. Thromb. Haemost..

[46] Abdel-Razeq H., Sharaf B., Al-Jaghbeer M.J., Abu-Fares H., Bater R., Shaer M.A., Abu-Jaish H., Laban D.A., Salamah O., Tamimi F. (2023). COMPASS-CAT versus Khorana risk assessment model for predicting venous thromboembolic events in patients with non-small cell lung cancer on active treatment with chemotherapy and/or immunotherapy, the CK-RAM study. J. Thromb. Thrombolysis.

[47] Song J., Morgan A.A., Ahn J., Li W.F., Chang Y., Chang Y.C., Fahimuddin M., Chi K.Y., Wu L.W., Chiang C.H. (2025). Validating Khorana Risk Score in gastric cancer patients on immune checkpoint inhibitors and chemotherapy. Immunotherapy.

[48] Xia X., Chen S., Huang C., Ye Y., Shen Y., Wang L. (2025). A new Score for Predicting Immune Checkpoint Inhibitor-Associated Thrombosis in Cancer Patients. Clin. Appl. Thromb. Hemost..

[49] Postow M.A., Sidlow R., Hellmann M.D. (2018). Immune-Related Adverse Events Associated with Immune Checkpoint Blockade. N. Engl. J. Med..

[50] Fawaz H., Numan H., El Charif M.H., Charbel N., El Khoury S., Rizkallah J., El Masri A., Tfayli A., Kreidieh F. (2025). Exploring the Emerging Association Between Immune Checkpoint Inhibitors and Thrombosis. J. Clin. Med..

[51] Bu D.X., Tarrio M., Maganto-Garcia E., Stavrakis G., Tajima G., Lederer J., Jarolim P., Freeman G.J., Sharpe A.H., Lichtman A.H. (2011). Impairment of the programmed cell death-1 pathway increases atherosclerotic lesion development and inflammation. Arterioscler. Thromb. Vasc. Biol..

[52] Wei S.C., Duffy C.R., Allison J.P. (2018). Fundamental Mechanisms of Immune Checkpoint Blockade Therapy. Cancer Discov..

[53] Zhang Y., Zhang Z., Wei R., Miao X., Sun S., Liang G., Chu C., Zhao L., Zhu X., Guo Q. (2020). IL (Interleukin)-6 Contributes to Deep Vein Thrombosis and Is Negatively Regulated by miR-338-5p. Arterioscler. Thromb. Vasc. Biol..

[54] Andrews L.P., Butler S.C., Cui J., Cillo A.R., Cardello C., Liu C., Brunazzi E.A., Baessler A., Xie B., Kunning S.R. (2024). LAG-3 and PD-1 synergize on CD8+ T cells to drive T cell exhaustion and hinder autocrine IFN-γ-dependent anti-tumor immunity. Cell.

[55] Sato R., Imamura K., Sakata S., Ikeda T., Horio Y., Iyama S., Akaike K., Hamada S., Jodai T., Nakashima K. (2019). Disorder of Coagulation-Fibrinolysis System: An Emerging Toxicity of Anti-PD-1/PD-L1 Monoclonal Antibodies. J. Clin. Med..

[56] Tabas I., Lichtman A.H. (2017). Monocyte-Macrophages and T Cells in Atherosclerosis. Immunity.

[57] Witztum J.L., Lichtman A.H. (2014). The influence of innate and adaptive immune responses on atherosclerosis. Annu. Rev. Pathol..

[58] Schalper K.A., Carleton M., Zhou M., Chen T., Feng Y., Huang S.P., Walsh A.M., Baxi V., Pandya D., Baradet T. (2020). Elevated serum interleukin-8 is associated with enhanced intratumor neutrophils and reduced clinical benefit of immune-checkpoint inhibitors. Nat. Med..

[59] Yuen K.C., Liu L.F., Gupta V., Madireddi S., Keerthivasan S., Li C., Rishipathak D., Williams P., Kadel E.E., Koeppen H. (2020). High systemic and tumor-associated IL-8 correlates with reduced clinical benefit of PD-L1 blockade. Nat. Med..

[60] Brinkmann V., Reichard U., Goosmann C., Fauler B., Uhlemann Y., Weiss D.S., Weinrauch Y., Zychlinsky A. (2004). Neutrophil extracellular traps kill bacteria. Science.

[61] Shim Y.J., Rayman P., Pavicic P., Swaidani S., Khorana A.A., Diaz-Montero M., McCrae K.R. (2022). Immune Checkpoint Inhibitors (ICI) Promote Neutrophil-Platelet Aggregate and NET Formation in Tumor-Bearing Mice. Blood.

[62] Lisman T. (2018). Platelet-neutrophil interactions as drivers of inflammatory and thrombotic disease. Cell Tissue Res..

[63] Wang Y., Braun O.Ö., Zhang S., Norström E., Thorlacius H. (2015). Monocytes regulate systemic coagulation and inflammation in abdominal sepsis. Am. J. Physiol. Heart Circ. Physiol..

[64] Bode M., Mackman N. (2014). Regulation of tissue factor gene expression in monocytes and endothelial cells: Thromboxane A2 as a new player. Vascul. Pharmacol..

[65] Theofilis P., Sagris M., Oikonomou E., Antonopoulos A.S., Siasos G., Tsioufis C., Tousoulis D. (2021). Inflammatory Mechanisms Contributing to Endothelial Dysfunction. Biomedicines.

[66] Gross P.L., Furie B.C., Merrill-Skoloff G., Chou J., Furie B. (2005). Leukocyte-versus microparticle-mediated tissue factor transfer during arteriolar thrombus development. J. Leukoc. Biol..

[67] Min J.K., Kim Y.M., Kim S.W., Kwon M.C., Kong Y.Y., Hwang I.K., Won M.H., Rho J., Kwon Y.G. (2005). TNF-related activation-induced cytokine enhances leukocyte adhesiveness: Induction of ICAM-1 and VCAM-1 via TNF receptor-associated factor and protein kinase C-dependent NF-kappaB activation in endothelial cells. J. Immunol..

[68] Suero-Abreu G.A., Zanni M.V., Neilan T.G. (2022). Atherosclerosis With Immune Checkpoint Inhibitor Therapy: Evidence, Diagnosis, and Management: JACC: CardioOncology State-of-the-Art Review. JACC CardioOncol..

[69] Daxini A., Cronin K., Sreih A.G. (2018). Vasculitis associated with immune checkpoint inhibitors-a systematic review. Clin. Rheumatol..

[70] Rivet V., Guillon B., Sibaud V., Dion J., Pastissier A., Delavigne K., Cougoul P., Rauzy O., Comont T. (2025). Case Report: Acral vasculitis induced by Immune Checkpoint Inhibitors: A case series and literature review. Front. Oncol..

[71] Ott P.A., Hodi F.S., Kaufman H.L., Wigginton J.M., Wolchok J.D. (2017). Combination immunotherapy: A road map. J. Immunother. Cancer..

[72] Ay C., Simanek R., Vormittag R., Dunkler D., Alguel G., Koder S., Kornek G., Marosi C., Wagner O., Zielinski C. (2008). High plasma levels of soluble P-selectin are predictive of venous thromboembolism in cancer patients: Results from the Vienna Cancer and Thrombosis Study (CATS). Blood.

[73] Khorana A.A. (2012). Cancer-associated thrombosis: Updates and controversies. Hematol. Am. Soc. Hematol. Educ. Program.

[74] Khorana A.A., Francis C.W., Culakova E., Kuderer N.M., Lyman G.H. (2008). Plasma tissue factor may be predictive of venous thromboembolism in pancreatic cancer. J. Thromb. Haemost..

[75] Ay C., Pabinger I., Cohen A.T. (2017). Cancer-associated venous thromboembolism: Burden, mechanisms, and management. Thromb. Haemost..

[76] Kuderer N.M., Ortel T.L., Francis C.W. (2009). Impact of venous thromboembolism and anticoagulation on cancer and cancer survival. J. Clin. Oncol..

[77] Papayannopoulos V. (2018). Neutrophil extracellular traps in immunity and disease. Nat. Rev. Immunol..

[78] Cedervall J., Zhang Y., Olsson A.K. (2016). Tumor-Induced NETosis as a Risk Factor for Metastasis and Organ Failure. Cancer Res..

[79] Demers M., Wagner D.D. (2014). NETosis: A new factor in tumor progression and cancer-associated thrombosis. Semin. Thromb. Hemost..

[80] Thålin C., Daleskog M., Goransson S.P., Schatzberg D., Lasselin J., Laska A.C., Kallner A., Helleday T., Wallén H., Demers M. (2017). Validation of an enzyme-linked immunosorbent assay for the detection of citrullinated histone H3 as a marker of neutrophil extracellular traps formation. J. Thromb. Haemost..

[81] Hu J.R., Florido R., Lipson E.J., Naidoo J., Ardehali R., Tocchetti C.G., Lyon A.R., Padera R.F., Johnson D.B., Moslehi J. (2019). Cardiovascular toxicities associated with immune checkpoint inhibitors. Cardiovasc. Res..

[82] Moik F., Riedl J.M., Barth D., Berton F., Fink M., Englisch C., Hoeller C., Fuereder T., Ay L., Pabinger I. (2024). Early Change in C-Reactive Protein and Venous Thromboembolism in Patients Treated With Immune Checkpoint Inhibitors. JACC CardioOncol..

[83] Qin S., Xu L., Yi M., Yu S., Wu K. (2019). Recent advances on anti-angiogenesis receptor tyrosine kinase inhibitors in cancer therapy. J. Hematol. Oncol..

[84] Awadalla M., Mahmood S.S., Groarke J.D., Hassan M.Z.O., Nohria A., Rokicki A., Murphy S.P., Mercaldo N.D., Zhang L., Zlotoff D.A. (2020). Global Longitudinal Strain and Cardiac Events in Patients With Immune Checkpoint Inhibitor-Related Myocarditis. J. Am. Coll. Cardiol..

[85] Riedl J., Preusser M., Nazari P.M., Posch F., Panzer S., Marosi C., Birner P., Thaler J., Brostjan C., Lötsch D. (2017). Podoplanin expression in primary brain tumors induces platelet aggregation and increases risk of venous thromboembolism. Blood.

[86] Takemoto A., Okitaka M., Takagi S., Takami M., Sato S., Nishio M., Okumura S., Fujita N. (2017). A critical role of platelet TGF-β release in podoplanin-mediated tumour invasion and metastasis. Sci. Rep..

[87] Skoulidis F., Byers L.A., Diao L., Papadimitrakopoulou V.A., Tong P., Izzo J., Behrens C., Kadara H., Parra E.R., Rodriguez Canales J. (2015). Co-occurring genomic alterations define major subsets of KRAS-mutant lung adenocarcinoma with distinct biology, immune profiles, and therapeutic vulnerabilities. Cancer Discov..

[88] Di Federico A., De Giglio A., Parisi C., Gelsomino F. (2021). STK11/LKB1 and KEAP1 mutations in non-small cell lung cancer: Prognostic rather than predictive?. Eur. J. Cancer.

[89] Dunbar A., Bolton K.L., Devlin S.M., Sanchez-Vega F., Gao J., Mones J.V., Wills J., Kelly D., Farina M., Cordner K.B. (2021). Genomic profiling identifies somatic mutations predicting thromboembolic risk in patients with solid tumors. Blood.

[90] Doubre H., Monnet I., Azarian R., Girard F., Meyer G., Trichereau J., Devillier P., Van Dreden P., Couderc L.J., Chouaid C. (2024). Plasma tissue factor activity in lung cancer patients predicts venous thromboembolism and poor overall survival. Res. Pract. Thromb. Haemost..

[91] Thaler J., Ay C., Mackman N., Metz-Schimmerl S., Stift J., Kaider A., Müllauer L., Gnant M., Scheithauer W., Pabinger I. (2013). Microparticle-associated tissue factor activity in patients with pancreatic cancer: Correlation with clinicopathological features. Eur. J. Clin. Investig..

[92] Rak J., Milsom C., May L., Klement P., Yu J. (2006). Tissue factor in cancer and angiogenesis: The molecular link between genetic tumor progression, tumor neovascularization, and cancer coagulopathy. Semin. Thromb. Hemost..

[93] Bronkhorst A.J., Ungerer V., Holdenrieder S. (2019). The emerging role of cell-free DNA as a molecular marker for cancer management. Biomol. Detect. Quantif..

[94] Nilsson R.J., Balaj L., Hulleman E., van Rijn S., Pegtel D.M., Walraven M., Widmark A., Gerritsen W.R., Verheul H.M., Vandertop W.P. (2011). Blood platelets contain tumor-derived RNA biomarkers. Blood.

[95] Best M.G., Wesseling P., Wurdinger T. (2018). Tumor-Educated Platelets as a Noninvasive Biomarker Source for Cancer Detection and Progression Monitoring. Cancer Res..

[96] Freitas-Dias C., Gonçalves F., Martins F., Lemos I., Gonçalves L.G., Serpa J. (2024). Interaction between NSCLC Cells, CD8+ T-Cells and Immune Checkpoint Inhibitors Potentiates Coagulation and Promotes Metabolic Remodeling-New Cues on CAT-VTE. Cells.

[97] Foley J.H., Conway E.M. (2016). Cross talk pathways between coagulation and inflammation. Circ. Res..

[98] Lyon A.R., Yousaf N., Battisti N.M.L., Moslehi J., Larkin J. (2018). Immune checkpoint inhibitors and cardiovascular toxicity. Lancet Oncol..

[99] Ay C., Dunkler D., Simanek R., Thaler J., Koder S., Marosi C., Zielinski C., Pabinger I. (2011). Prediction of venous thromboembolism in patients with cancer by measuring thrombin generation: Results from the Vienna Cancer and Thrombosis Study. J. Clin. Oncol..

[100] Li A., La J., May S.B., Guffey D., da Costa W.L., Amos C.I., Bandyo R., Milner E.M., Kurian K.M., Chen D.C.R. (2023). Derivation and validation of a clinical risk assessment model for cancer associated thrombosis in two unique US health care systems. J. Clin. Oncol..

[101] Khorana A.A. (2010). Venous thromboembolism and prognosis in cancer. Thromb. Res..

[102] Patell R., Rybicki L., McCrae K.R., Khorana A.A. (2017). Predicting risk of venous thromboembolism in hospitalized cancer patients: Utility of a risk assessment tool. Am. J. Hematol..

[103] Björklund J., Rautiola J., Zelic R., Edgren G., Bottai M., Nilsson M., Vincent P.H., Fredholm H., Falconer H., Sjövall A. (2024). Risk of venous thromboembolic events after surgery for cancer. JAMA Netw. Open.

[104] Lyman G.H., Eckert L., Wang Y., Wang H., Cohen A. (2013). Venous thromboembolism risk in patients with cancer receiving chemotherapy: A real-world analysis. Oncologist.

[105] Lyman G.H., Carrier M., Ay C., Di Nisio M., Hicks L.K., Khorana A.A., Leavitt A.D., Lee A.Y.Y., Macbeth F., Morgan R.L. (2021). American Society of Hematology 2021 guidelines for management of venous thromboembolism: Prevention and treatment in patients with cancer. Blood Adv..

[106] Bosch F.T.M., Mulder F.I., Kamphuisen P.W., Middeldorp S., Bossuyt P.M., Büller H.R., van Es N. (2020). Primary thromboprophylaxis in ambulatory cancer patients with a high Khorana score: A systematic review and meta-analysis. Blood Adv..

[107] Alexander M., Harris S., Underhill C., Torres J., Sharma S., Lee N., Wong H.L., Eek R., Michael M., Tie J. (2023). Risk-directed ambulatory thromboprophylaxis in lung and gastrointestinal cancers: The TARGET-TP randomized clinical trial. JAMA Oncol..

[108] Liu M., Wang G., Li Y., Wang H., Liu H., Guo N., Han C., Peng Y., Yang M., Liu Y. (2020). Efficacy and safety of thromboprophylaxis in cancer patients: A systematic review and meta-analysis. Ther. Adv. Med. Oncol..

[109] Karamouzis M.V., Athanasiadis I., Samelis G., Vallilas C., Bokas A., Nikolaidi A., Dimitriadou A., Sarantis P., Pistamaltzian N., Schizas D. (2021). The impact of thromboprophylaxis on the survival of patients with advanced pancreatic cancer. The pancreatic cancer and tinzaparin (PaCT) study. Cancers.

[110] Hoemme A., Barth H., Haschke M., Krähenbühl S., Strasser F., Lehner C., von Kameke A., Wälti T., Thürlimann B., Früh M. (2019). Prognostic Impact of Polypharmacy and Drug Interactions in Patients with Advanced Cancer. Cancer Chemother. Pharmacol..

[111] Steffel J., Collins R., Antz M., Cornu P., Desteghe L., Haeusler K.G., Oldgren J., Reinecke H., Roldan-Schilling V., Rowell N. (2021). 2021 European Heart Rhythm Association Practical Guide on the Use of Non-Vitamin K Antagonist Oral Anticoagulants in Patients with Atrial Fibrillation. Europace.

[112] Peixoto de Miranda É.J.F., Takahashi T., Iwamoto F., Yamashiro S., Samano E., Macedo A.V.S., Ramacciotti E. (2020). Drug-Drug Interactions of 257 Antineoplastic and Supportive Care Agents with 7 Anticoagulants: A Comprehensive Review of Interactions and Mechanisms. Clin. Appl. Thromb. Hemost..

[113] Van der Hulle T., den Exter P.L., van den Hoven P., van der Hoeven J.J., van der Meer F.J., Eikenboom J., Huisman M.V., Klok F.A. (2016). Cohort Study on the Management of Cancer-Associated Venous Thromboembolism Aimed at the Safety of Stopping Anticoagulant Therapy in Patients Cured of Cancer. Chest.

[114] Barca-Hernando M., Lopez-Ruz S., Marin-Romero S., Garcia-Garcia V., Elias-Hernandez T., Otero-Candelera R., Carrier M., Jara-Palomares L. (2023). Risk of recurrent cancer-associated thrombosis after discontinuation of anticoagulant therapy. Res. Pract. Thromb. Haemost..

[115] Mahé I., Carrier M., Mayeur D., Chidiac J., Vicaut E., Falvo N., Sanchez O., Grange C., Monreal M., López-Núñez J.J. (2025). Extended Reduced-Dose Apixaban for Cancer-Associated Venous Thromboembolism. N. Engl. J. Med..

[116] Piazza G., Bikdeli B., Pandey A.K., Krishnathasan D., Khairani C.D., Bejjani A., Morrison R.H., Hogan H., Rashedi S., Pfeferman M. (2025). Apixaban for Extended Treatment of Provoked Venous Thromboembolism. N. Engl. J. Med..

[117] Ay C., Vormittag R., Dunkler D., Simanek R., Chiriac A.L., Drach J., Quehenberger P., Wagner O., Zielinski C., Pabinger I. (2009). D-dimer and prothrombin fragment 1 + 2 predict venous thromboembolism in patients with cancer: Results from the Vienna Cancer and Thrombosis Study. J. Clin. Oncol..

[118] Vladic N., Englisch C., Berger J.M., Moik F., Hayden H., Thaler J., Berghoff A.S., Fuereder T., Preusser M., Pabinger I. (2025). Longitudinal dynamics of hemostatic biomarkers in patients with cancer receiving immune checkpoint inhibitors vs chemotherapy: Results from the Vienna CAT-BLED study. Res. Pract. Thromb. Haemost..

