# Supplementary material for: Cancer-Associated Thrombosis in Patients Treated with Immune Checkpoint Inhibitors
Source: Int J Mol Sci. 2026 Feb 15;27(4):1874. doi: 10.3390/ijms27041874 (PMC12941293; doi:10.3390/ijms27041874)
Supplement: Supplementary file 1 [file ijms-27-01874-s001.zip › ijms-4112828-supplementary.pdf]

Table S1. Schematic representation of a conceptual, trajectory-based framework integrating major biomarkers under investigation for their potential association with ICI–related thrombosis. The model is intended for hypothesis generation and future validation, not for routine clinical decision-making. CRP, C-reactive protein; NET, neutrophil extracellular trap; EV, extracellular vesicles; TF, tissu factor.

Biomarker	Timing relative to ICIs	Quantitative evidence in ICI cohorts	Assay standardization	Clinical readiness
**D-dimer**	Baseline and early post-ICI changes	No consistent ICI-specific HRs; associations mainly descriptive	High	Exploratory / adjunctive
**CRP (CRP flare)**	Early increase (4–6 weeks)	HR 2–3 reported in selected cohorts	High	Emerging
**Soluble P-selectin**	Not systematically defined	No validated effect sizes in ICI cohorts	Moderate	Exploratory
**NET-related markers**	Early inflammatory phase	Lacking quantitative ICI-specific data	Low	Research only
**EV-associated TF**	Advanced disease context	No quantitative data in ICI-treated patients	Low	Research only
